# Effects of Long-Term Selection in the Border Collie Dog Breed: Inbreeding Purge of Canine Hip and Elbow Dysplasia

**DOI:** 10.3390/ani10101743

**Published:** 2020-09-25

**Authors:** Virág Ács, György Kövér, János Farkas, Árpád Bokor, István Nagy

**Affiliations:** 1 Department of Animal Nutrition, Szent István University Kaposvár Campus, 40, Guba S. str., H-7400 Kaposvár, Hungary; 2 Department of Mathematics and Informatics, Szent István University Kaposvár Campus, 40, Guba S. str., H-7400 Kaposvár, Hungary; kover.gyorgy@ke.hu (G.K.); farkas.janos@ke.hu (J.F.); 3 Department of Hippology, Szent István University Kaposvár Campus, 40, Guba S. str., H-7400 Kaposvár, Hungary; bokor.arpad@ke.hu; 4 Department of Animal Science, Szent István University Kaposvár Campus, 40, Guba S. str., H-7400 Kaposvár, Hungary; nagy.istvan@ke.hu

**Keywords:** border collie, inbreeding, purging, hip dysplasia, elbow dysplasia

## Abstract

**Simple Summary:**

For dog breeders, health is one of the main criteria when choosing a breeding animal; thus, selection for good anatomy is the key to reduce orthopedic disorders. In many dog breeds, radiographic screening for canine hip and elbow dysplasia is a compulsory test for breeding; however, these multifactorial traits are determined by genetic and environmental factors. Therefore, it is extremely difficult to eliminate these disorders from the population. In natural selection, such traits can be “purged” out of the population with inbreeding. The study aimed to examine the inbreeding-purge of canine hip and elbow dysplasia in the border collie breed. The main conclusion was that over-representation of homozygous individuals may have a positive effect on hip and elbow conformation.

**Abstract:**

Pedigree data of 13,339 border collie dog was collected along with canine hip dysplasia (CHD) and canine elbow dysplasia (CED) records (1352 CHD and 524 CED), and an inbreeding–purging (IP) model was created. Ancestral inbreeding coefficients were calculated by using a gene dropping simulation method with GRain 2.2 software. Cumulative logit models (CLM) for CHD and CED were fitted using a logit-link Poisson distribution and the classical (F__W_), and ancestral inbreeding (F__BAL_, F__KAL_, and F__KAL_NEW_) coefficients as linear regression coefficients. The effective population size was calculated from F__W_ and decreased in the examined period along with an increase of F__W_; however, slight differences were found as a consequence of breeding dog imports. CHD values were lowered by the expansion of F__BAL_, as the alleles had been inbred in the past. For CHD, signs of purging were obtained. There was a positive trend regarding the breeding activity (both sire and dam of the future litters should be screened and certified free from CHD and CED), as years of selection increased the frequency of alleles with favorable hip and elbow conformation. Division of the ancestral inbreeding coefficient showed that alleles that had been identical by descent (IBD) for the first time (F__KAL_NEW_) had a negative effect on both traits, while F__KAL_ has shown favorable results for alleles IBD in past generations. Some authors had proven this phenomenon in captive populations or experimental conditions; however, no evidence of inbreeding purge has ever been described in dog populations. Despite the various breeding practices, it seems that alleles of these polygenic disorders could be successfully purged out of the population with long-term selection.

## 1. Introduction

Dog breeds had gone through several morphological and functional changes over the centuries as a result of selective breeding. In the 19th century—at the time of Kennel Club foundations—dog populations went through the greatest bottleneck effect in dog breeding history. With the rising interest in purebred dogs, selection pressure has affected dog breeds considerably, and they have suffered a higher loss of genetic diversity than other domesticated species [1,2] also reported that out of 207 examined breeds, collies showed the highest average inbreeding. Due to the repeated use of popular sires, breeding for phenotype and linebreeding also resulted in smaller effective population size [3]. While undesirable traits are eliminated from other agricultural species as breeding programs focus on production and longevity, selection in dog breeding concentrates on looking and behaving in certain ways [4] Alongside the transformation of breed functions (from working dogs to companion pets), lower within-breed heterogeneity and unhealthy anatomy leads to inherited disorders, which can be connected to breed standards highlighting show ring appearance. Some of these health problems are monogenic (produced by a single gene or allele); on the other hand, there are multifactorial traits that are influenced by genetic factors and the environment. 

The border collie was mainly a working breed during the last two centuries; however, nowadays show-line dogs make up a great part of the population as a good family pet with lower energy levels. Dog breeders and the different kennel clubs operate with compulsory genetic and clinical health tests for breeding animals (in Hungary, compulsory tests are only in the working line) to increase the chance to produce healthy offspring. The most common clinical tests for orthopedic disorders are the screening of possible canine hip dysplasia (CHD) and canine elbow dysplasia (CED). These multifactorial traits are both affected by genetic and environmental components showing great incidence variability among breeds with mixed results of phenotypic selection [5,6]. 

CHD-affected dogs diagnosed by radiographic imaging have abnormal hip development with femoral head luxation and ossification delay [7]. Selection is based on phenotype by scaling images from normal to severe [8]. CED was previously defined as a combination of orthopedic disorders of the foreleg, such as fragmented medial coronoid process, osteochondritis, an incongruity of the elbow joint, and ununited anconeal process [9] leading to osteoarthritis, which is debilitating and incurable. As a result, dysplasia categorized as severe or moderate is painful and frequently causes lameness.

Breeding schemes showed a diverse degree of improvement in hip and elbow joint confirmation due to different sample sizes assessment protocols [6,10] and variation in the effectiveness of selection. CHD was registered to Orthopedic Foundation of Animals (OFA) in the ’60s, to provide data for breeding programs by integrating genetic and phenotypic information of animals and support selection decisions [11,12]. Heritability of CHD ranges from h2 = 0.35 [6] to 0.58 [13,14], while CED heritability ranges from 0.01 to 0.36 [15], depending on the pedigree completeness and breed differences. In closed populations, such as purebred dog populations, selection pressure and inbreeding may reduce fitness since inbreeding enhances the number of homozygotes of a certain allele (inbreeding depression). In conservation genetics, [16] proposed that N_e_ should be at least 100 to avoid short-term inbreeding depression; this concept should be also be promoted in dog breeding. Nevertheless, inbreeding promotes the expression of recessive alleles; it also gives a rise to the effectiveness of natural selection known as genetic “purge” [17]. The beneficial effects of purging were first reported in a small captive Speke’s gazelle population [18,19] where the population’s reproductive performance was improved within few generations. According to [18,19] selection and inbreeding were combined to get rid of the deleterious alleles. Purging can be effective when the average effect of deleterious mutations is strong (relative to the effective population size); inbreeding occurs gradually and over several generations, and the population is sufficiently isolated so that purged deleterious alleles are not reintroduced by immigration [20]. Although purging has extensively been analyzed, research conducted in domesticated species is rare, and it is mostly related to a few cattle populations [21,22].

The study aimed to examine the border collie breed in an inbreeding–purging (IP) concept for CHD and CED with the model of [23]. This concept uses ancestral inbreeding in an attempt to demonstrate that inbreeding depression is partially purged due to selection.

## 2. Materials and Methods

### 2.1. Data Collection

The database of the examined population contained 13,339 individuals (5649 males and 7750 females) built up from electronic herd books and pedigrees from Hungarian breeders. The reference population consisted of 1877 border collies (929 males and 948 females) born between 1990 and 2016 with relevant CHD and CED data. Genealogy information was tracked back from the late 1800s to the present day. Records were created with EquiHun Pedigree Builder [24] with the following parameters:

- Individual identity number;
- Male parent;
- Female parent;
- Date of birth;
- Country of birth (i.e., country of origin).

The reference population had pedigree completeness of 99.6% up to 15 generations, and the pedigree analysis was carried out by [25]. Ancestral inbreeding coefficients were calculated by a gene dropping simulation method [26,27] with GRain 2.2 (Wageningen University, [28]) to avoid overestimation of ancestral inbreeding. In the present study, 1,000,000 simulations were used, and correlations between all inbreeding coefficients were tested. 1352 CHD and 525 CED data were added to pedigrees for further evaluation. General requirements of CHD and CED screening were described in detail by FCI (Federation Cynologique Internationale, [29]), where the main regulations were as follows:

- The minimum age of the dog for radiographic imaging is 1 year;
- The dog must be identified by a microchip;
- All dogs should be sufficiently sedated or anesthetized during the procedure to relax all muscles.

The categories of CHD data were summarized in Table 1. For further evaluation, FCI categories were coded with numbers from 0 (excellent) to 4 (severe). Radiographic images were taken individually by veterinarians and sent for evaluation to the Pet Orthopedic Association Hungary for uniform assessment.

CED categories are described in Table 2 and coded from 0 (normal) to 3 (severe).

### 2.2. Data Analysis

The applied models combine the genetic basis of inbreeding depression with a purging mechanism, based on the assumption that inbred animals with inbred ancestry are less responsive to inbreeding depression than inbred animals with non-inbred ancestry [19]. Since the inbred ancestors that can attain breeding requirements for health are less likely to be carriers of deleterious alleles, inbreeding depression must be present for purging. 

The change of inbreeding depression due to purging was calculated based on [30] as follows:

*u* = *u*_0_ + *β*_*f*_*f* + *β*_*fa*_*fa* + *β*_*fd*_*fd* + *β*_*YOB*_*YOB*

where where *u* is the logit transformation of a measure of fitness (CHD or CED score); *u*_0_ is the mean fitness of non-inbred animals; *f_a_* is the ancestral inbreeding coefficient; and *β_f_*, *βfa, βfd* and *β_YOB_* are the regression coefficients associated with the inbreeding coefficient (*f*), the interaction term *ff_a_*, maternal inbreeding (*f_d_)* and year of birth (*YOB*), respectively. 

The inbreeding coefficients were summarized by birth year (1990–2016), from the first date of radiographic examination. The models contained the following inbreeding coefficients as logistic regression coefficients:

- F__W_: Inbreeding coefficient described by [31].
- F__BAL_: Ancestral inbreeding coefficient, determined as the cumulative proportion of the genome exposed to inbreeding effects [26]. F_BAL was created to test the magnitude and effectiveness of inbreeding depression as the extent to which individual’s ancestors had been subjected to inbreeding.
- F__KAL_: Inbreeding coefficient defined by [17]. The probability that alleles had been autozygous (IBD) in the previous generation at least once, where the common ancestor was presented on both sides of the pedigree.
- F__KAL_NEW_: Kalinowski new inbreeding coefficient, described as alleles IBD, was inbred for the first time [17].

Effect plots of the applied models were created with the “effects” package in R [32] to display differences between the models and illustrate the IP, where purging is the fitness decline with increasing inbreeding (purifying selection, facilitated by inbreeding) where inbred animals with good performance have been selected from the population as parents, while the poorly performing inbred animals are not selected [22]. 

R package ‘’MASS’’ was used (Springer, New York, NY, USA [33]) to make a cumulative logit model (CLM) for ordinal responses. The data were fitted with ‘’polr’’ function. An effective population size was calculated from an individual increase of inbreeding (F__W_) [34]. 

To estimate the proportion of each dog’s genome that alleles were identical by descent in an ancestor of a dog, for the first time a stochastic approach was applied by gene dropping [35] with GRain 2.2 (Wageningen University, [28]), due to the fact that F_w and F__BAL_ are dependent. So, the procedure included two unique alleles assigned to each founder and generated the genotypes of all offspring along with the pedigree by Mendelian segregation rules. 

The structure of the applied models are described in Table 3 by the method of [36], as follows:

## 3. Results

**Figure 1:**
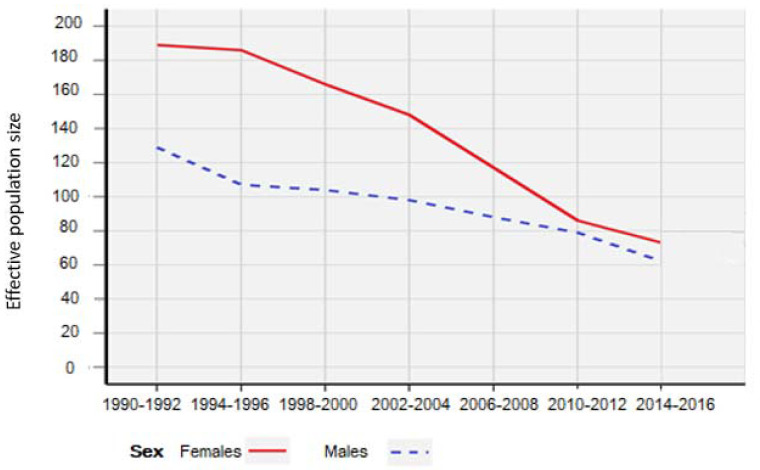
The effective population size of the border collie breed.

The effective population size of the border collie breed in the examined period (from the first litter born in Hungary until the present day) is demonstrated in Figure 1. 

The effective size of both male and female dogs decreased each year. Despite the popularity of the breed, owners usually buy cheaper dogs from unregistered breeders, and the number of dogs with registered pedigree diminishes each year. Besides, using favorite males for matings is a common trend in dog breeding that can lower the population size and enhance inbreeding. On the contrary, it can increase litter homogeneity, which is highly preferred from the breeder’s point of view. 

**Figure 2:**
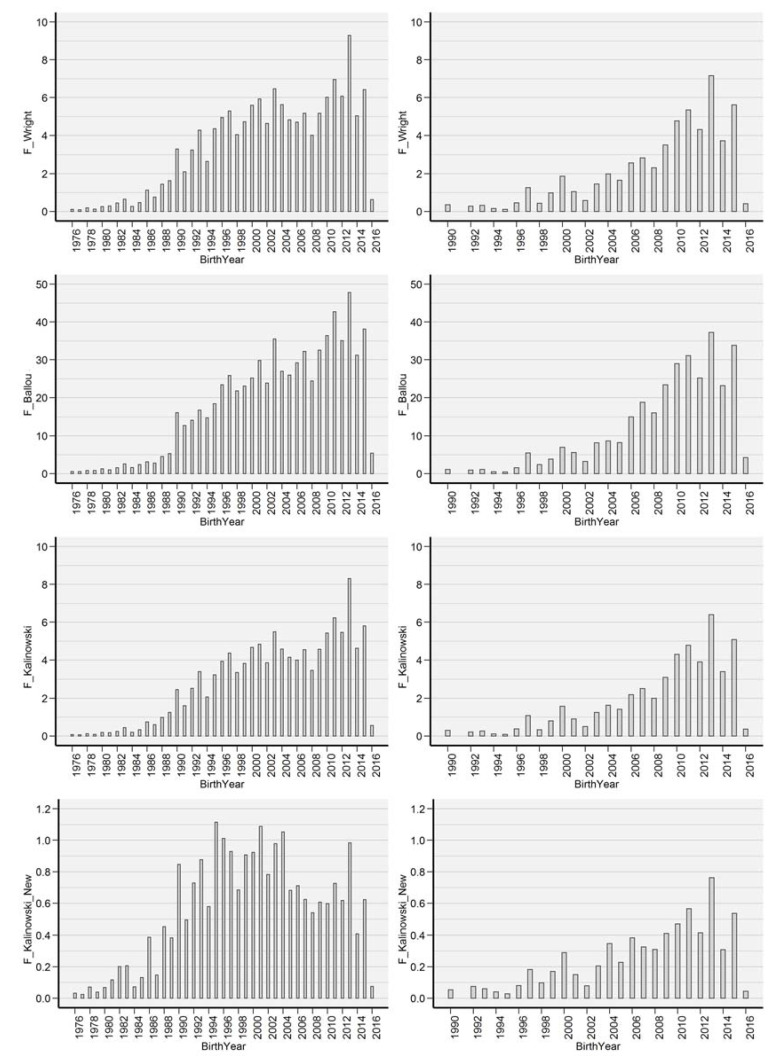
The estimated ancestral inbreeding coefficients by birth year in the total (left) and the reference (right) populations.

Figure 2 summarizes the estimated values for the ancestral inbreeding coefficients and F__W_ by birth year.

An increasing trend of estimated F__W_ values can be observed; however, differences were found between the examined years, thanks to the popularity of the working line from time to time. These dogs were imported mainly for FCI collecting style herding events. This finding indicates that differences between breeding trends can maintain genetic variability; on the other hand, the number of working-line border collies is still low compared to the show-line dogs [25]. Looking at the estimated values for F__BAL_ coefficients, similar tendencies were observed. The first border collie arrived in Hungary in 1990; thus, ancestral inbreeding was enhanced as the first kennels started their breeding programs. Table 4 summarizes the significant inbreeding coefficients regarding CED and CHD for the applied models.

For CHD, only F__KAL_NEW_ has no significant effect, while F__W_ and F__BAL_ in ‘’model 1’’ are insignificant for the development of CED in the population. Correlation coefficients of F__W_ and F__BAL_ are relatively weak (0.48), while those between F__KAL_ and F__W_ show a strong correlation (0.9) because of the part–whole relationship between them. Similar results were previously reported by [22], who examined the IP concept in the Irish Holstein–Friesian population.

### 3.1. Results for Purging in Canine Hip Dysplasia

**Figure 3:**
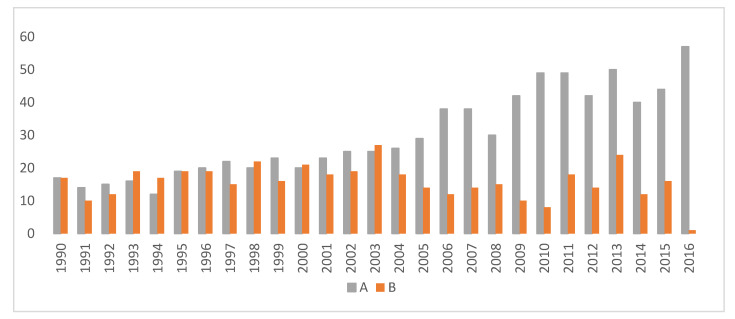
Number of excellent (**A**) and borderline (**B**) canine hip dysplasia (CHD) results in the reference population. **A**: Excellent; **B**: Borderline.

Figure 3 demonstrates the number of excellent (A) and borderline (B) CHD examinations by birth year.

Results show that the number of dogs with excellent results increased each year. Altogether, 1235 border collies were diagnosed with A and B hip results, while cases of mild, moderate and severe CHD were low (34).

**Figure 4:**
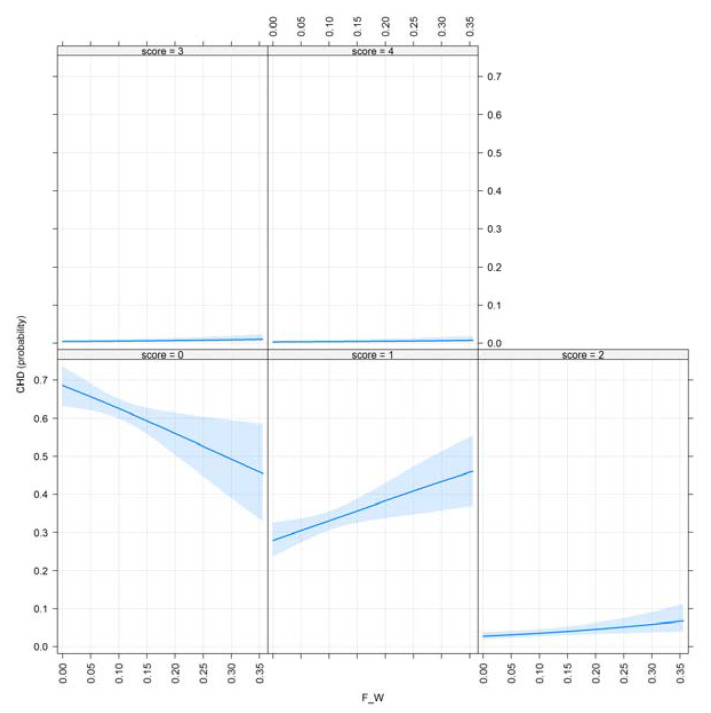
Effect plot of F__W_ for CHD, where scores from 0–4 illustrate hip results from A to E.

Figure 4 demonstrates the effect plots of the inbreeding–purging concept of variables for CHD and visualizes F__W_ showing their effect on the examined trait.

The shaded area represents a pointwise confidence band for the fitted values, based on standard errors computed from the covariance matrix of the fitted coefficients. The rug plot shows the location of the values of inbreeding. It is visible that as the inbreeding coefficient (F__W_) increases year-by-year in the population, the probability of dogs with excellent results increases; however, the genetic load of partially deleterious alleles is still represented in the population (score: 1–2).

Inbreeding in the ancestral population displays the phenomenon of purging. As F__BAL_ values increase, the probability of CHD decreases. It is detectable that after an initial drop, the examined inbred population recovered its level of health, thus with the occurrence of purging through several generations, it should contain fewer deleterious alleles. There is a great number of dogs with high ancestral inbreeding having excellent hip results (score: 0), while the probability of having ‘’borderline’’ or ‘’mild’’ hip results (score: 1–2) remains low. This may be the consequence of long-term selection for healthy hips as a favorable trait. 

The ancestral inbreeding coefficient defined by [17] showed similar tendencies to F__BAL_. ‘’Moderate’’ and ‘’severe’’ hip conditions remained constant regarding all significant inbreeding coefficients.

**Figure 5:**
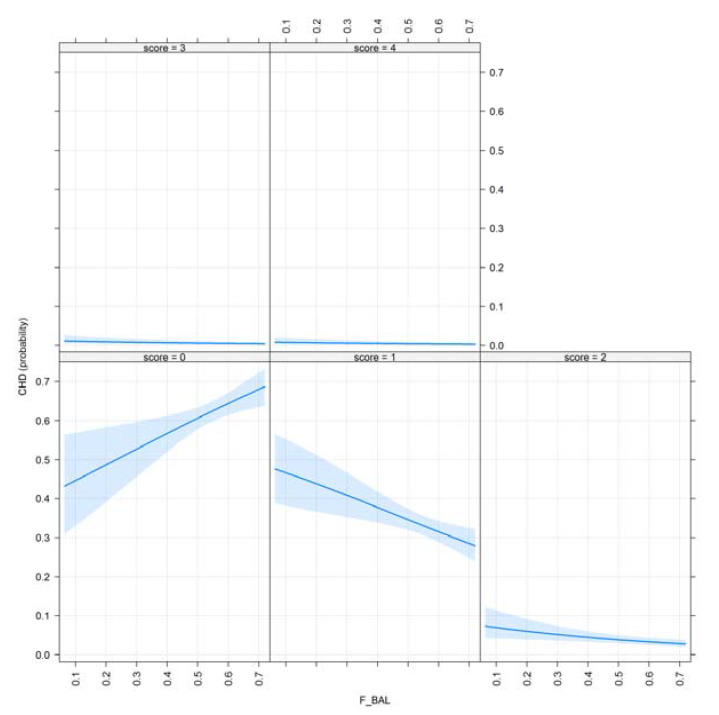
Effect plot of F__BAL_ for CHD.

**Figure 6:**
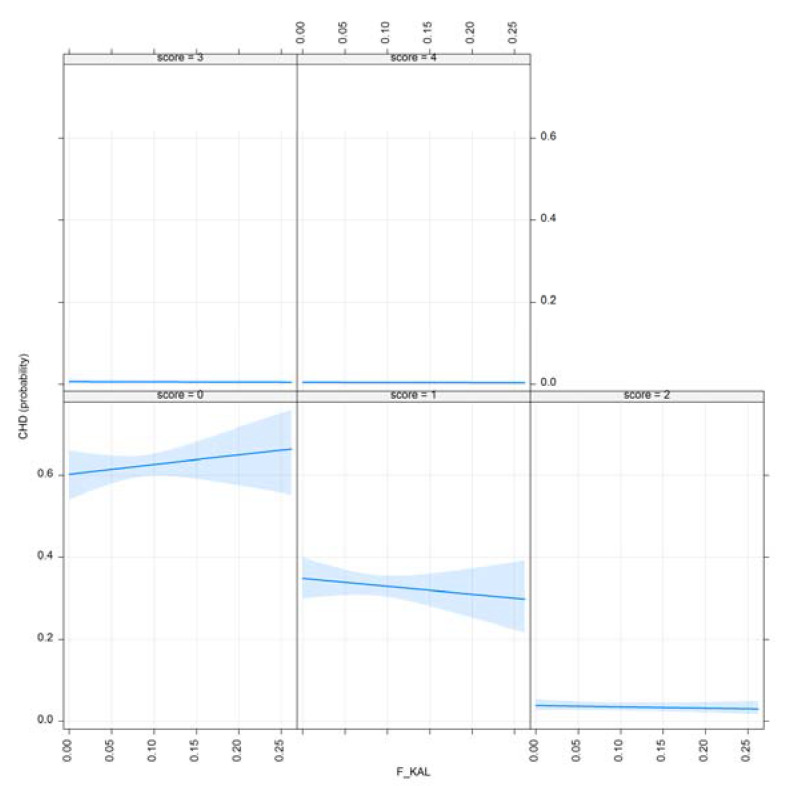
Effect plot of F__KAL_ for CHD.

Figure 5 and Figure 6 demonstrate the effect plots for F__KAL_ and F__KAL_NEW._

### 3.2. Results of Purging in Canine Elbow Dysplasia

For CED results, the tendencies were similar; however, the estimated values of classical inbreeding coefficient and the ancestral inbreeding defined by [26] did not show any significant differences (*p* = 0.802, *p* = 0.425). 

**Figure 7:**
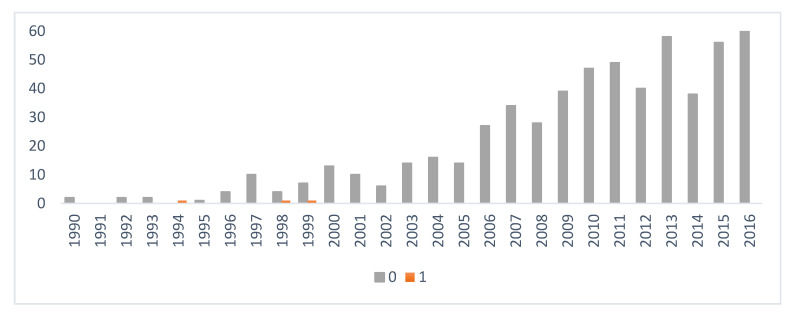
Number of normal (0) and slight (1) canine elbow dysplasia (CED) results by birth year in the reference population. 0: Normal; 1: Slight.

The number of normal (0) and slight (1) CED results is represented in Figure 7.

Normal elbow records were the most frequent during the studied period (5), while the rate of X-ray evaluations increased. Besides, the breeding strategies were advantageous, since only 27 dogs were diagnosed with severe CED and failed as a breeding animal. After the division of the inbreeding coefficient into two parts—F__KAL_ and F__KAL_NEW_—for CED shows, that selective breeding was successful. 

**Figure 8:**
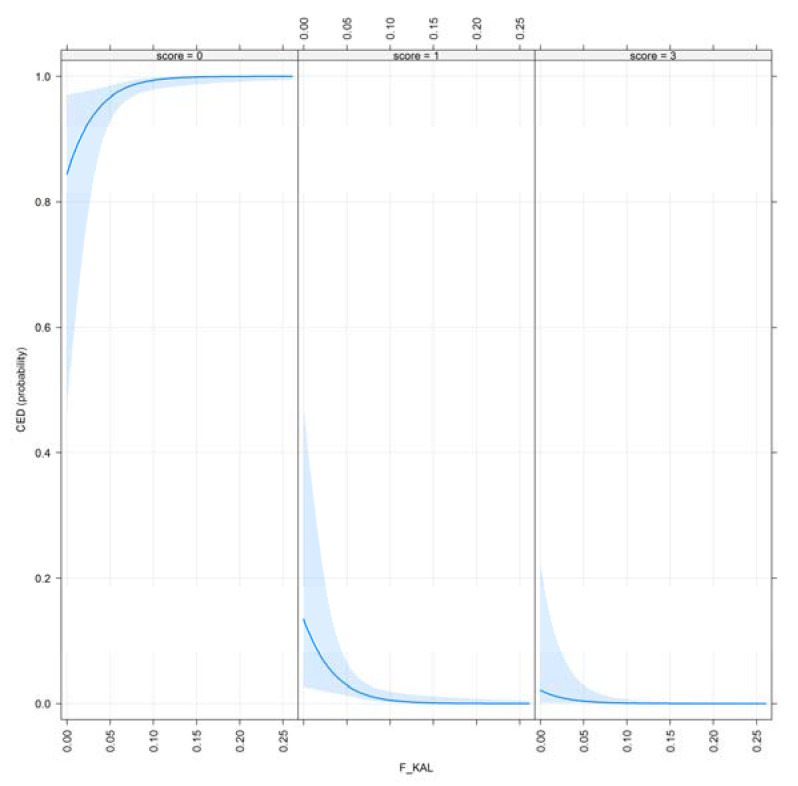
Effect plot of F__KAL_ for CED.

**Figure 9:**
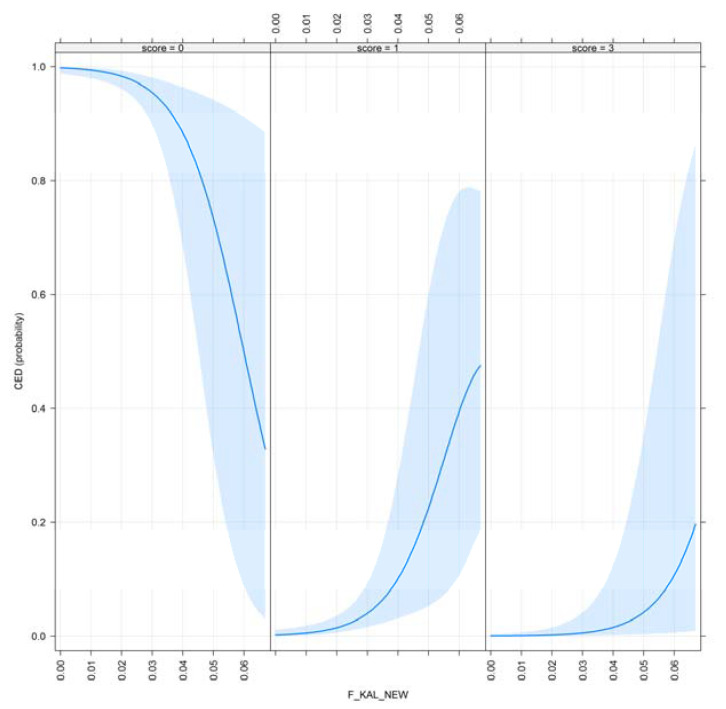
Effect plot of F__KAL_NEW_ for CED.

The effect plots for F__KAL_ and F__KAL_NEW_ are shown in Figure 8 and Figure 9.

CED for selection criteria are very recent (radiographic imaging to detect this disorder started in the mid-2000s in Hungary), and still optional. It can be concluded that as the amount of the ancestral inbreeding rises, CED results improve. This result is also favorable; however, only 2 out of these 525 scanned dogs had severe CED. 

These results demonstrate the differences between Ballou’s and Kalinowski’s concept. In this case, examining the estimated values for purging requires not just the classical inbreeding-purging concept, but the previously described ancestral inbreeding approaches.

## 4. Discussion

Within-breed variation was previously described by [37] and [1], showing that dog populations had a great selection pressure and several bottlenecks. Overuse of popular sires and a large amount of unequally used breeding animals [2] decrease genetic diversity. Nevertheless, other mating trends such as “outbreeding” and “outcrossing” may have a positive effect on genetic diversity and inbreeding depression [3]. Thus, consequences in connection with the health status of the breed can be divided into two categories: alleles concerning lethal and sub-lethal mutations and mildly deleterious mutations that are only partially recessive [38]. The over-represented homozygous individuals might have a positive effect, since recessive alleles can be purged out of the population [39,40] by the increased amount of inbreeding and selection, having a positive effect on traits in connection with health. Our results show that ancestral inbreeding coefficients had a positive effect on hip and elbow conformation; however, the genetic load was not completely excluded from the population. 

Comparing the inbreeding coefficients by [17], it can be concluded that as the number alleles IBD in the past increased, the hip conformation results started to improve. Before the availability of phenotypic assessments, there was no possibility to pre-select breeding dogs by their anatomical values. On the other hand, alleles IBD for the first time tended to have a negative effect on health. F__KAL_NEW_ tended to be higher, and the number of dogs with excellent and good hip results was enhanced. F__KAL_NEW_ was used only in a few studies [21,22] where the detrimental effects of these coefficients were reported for most of the examined traits. [41] reported the harmful effects of new inbreeding and the lack of negative effects of old inbreeding for reproductive traits in rabbits. 

The phenotypic trend for CHD and CED in 60 different dog breeds showed substantial differences according to [14]. The study pointed out that out of the examined breeds, border collies are not in the group of breeds that are highly affected by orthopedic disorders. According to [42], the correlation between radiographic and physical signs depends on physical demands (working dogs and family pets are different due to muscularity), age, and the breed. [43] previously reported a positive effect of selective breeding for exercise physiology and selective sweeps linked to genes influencing muscle fiber formation in thoroughbred horses. [44,45] and [46,47] also found that age at the X-ray examination has a serious impact on hip and elbow results. 

The most often cases for purging are slow inbreeding and competitive conditions [48,49]. As our models proved, recessive deleterious alleles seemed to be purged in inbred ancestors; thus, dogs with higher F__BAL_ and F__KAL,_ for both traits are expected to carry less of these alleles to the next generation than individuals with the same level of inbreeding but lower ancestral inbreeding values. [23] previously described this phenomenon in captive populations.

## 5. Conclusions

The decrease of effective population size points to a trend: that dog owners do not prefer to buy from registered breeders. The results show that models containing the alternative inbreeding coefficients and the significant positive effects of ancestral inbreeding coefficients on the examined traits suggest that the border collie population in Hungary experienced purging. This finding was not surprising when taking into account the long and complete pedigree and the slow but continuous inbreeding rate, and the very high ancestral inbreeding coefficient at the end of the analyzed period. 

To maintain variability, the genetic contribution of some preferred males could be limited by mating schemes to help the breeders with breeding decisions. Import breeding dogs could be also a solution to this problem; on the other hand, breeding standards are slightly different between countries, so this requires collaboration between breeding organizations and scientists to improve the health of the next generation over looks. 

## Figures and Tables

**Table 1:** Categories and code numbers of canine hip dysplasia.

Hip Rating	Category Name	Hip Scores
A	Excellent	0
B	Borderline	1
C	Mild	2
D	Moderate	3
E	Severe	4

**Table 2:** Elbow rating categories.

Elbow Rating	Category
0	Normal: No sign of arthrosis
1	Slight: Osteophytes, less than 2 mm
2	Medium: Osteophytes from 2 to 5 mm
3	Severe: Osteophytes, more than 5 mm

**Table 3:** Structure and fitting of the applied models.

Model	Component Models	AIC	
		CED	CHD
1	F__w_ + F__BAL_	95.56	2243.45
2	F__KAL_ + F__KAL_New_	92.82	2250.16

F__W_: inbreeding coefficient of the population; F__BAL_: ancestral inbreeding coefficient of the population; F__KAL_: Kalinowski inbreeding coefficient of the population; F__KAL_NEW_: Kalinowski “new” inbreeding coefficient of the population; AIC: akaike information criterion for model 1 and 2; CED: canine elbow dysplasia; CHD: canine hip dysplasia.

**Table 4:** Effect of the inbreeding coefficients for the examined traits.

Variables	CED	CHD
	Pr(>|z|)	Pr(>|z|)
F__W_	0.802	0.009 **
F__BAL_	0.425	0.003 **
F__KAL_	0.001 ***	0.003 **
F__KAL_NEW_	0.011 *	0.444

*: *p* ≤ 0.05, **: *p* ≤ 0.01, ***: *p* ≤ 0.001.
